# Comparative Study on Simulated Outdoor Navigation for Agricultural Robots

**DOI:** 10.3390/s24082487

**Published:** 2024-04-12

**Authors:** Feeza Khan Khanzada, Elahe Delavari, Woojin Jeong, Young Seek Cho, Jaerock Kwon

**Affiliations:** 1 Department of Electrical and Computer Engineering, University of Michigan-Dearborn, 4901 Evergreen Road, Dearborn, MI 48128-2406, USA; feezakk@umich.edu (F.K.K.); elahed@umich.edu (E.D.); jrkwon@umich.edu (J.K.); 2 WApplE Cloud Co., Ltd., #912, 313 Teheran-ro, Gangnam-gu, Seoul 06151, Republic of Korea; woojin@wapplecloud.com; 3 Department of Electronic Engineering, Wonkwang University, 460 Iksan-daero, Iksan-si 54538, Republic of Korea

**Keywords:** SLAM, behavior cloning, agricultural robotics, deep neural networks

## Abstract

This research presents a comprehensive comparative analysis of SLAM algorithms and Deep Neural Network (DNN)-based Behavior Cloning (BC) navigation in outdoor agricultural environments. The study categorizes SLAM algorithms into laser-based and vision-based approaches, addressing the specific challenges posed by uneven terrain and the similarity between aisles in an orchard farm. The DNN-based BC navigation technique proves efficient, exhibiting reduced human intervention and providing a viable alternative for agricultural navigation. Despite the DNN-based BC navigation approach taking more time to reach its target due to a constant throttle limit for steady speed, the overall performance in terms of driving deviation and human intervention is notable compared to conventional SLAM algorithms. We provide comprehensive evaluation criteria for selecting optimal techniques for outdoor agricultural navigations. The algorithms were tested in three different scenarios: Precision, Speed, and Autonomy. Our proposed performance metric, *P*, is weighted and normalized. The DNN-based BC algorithm showed the best performance among the others, with a performance of 0.92 in the *Precision* and Autonomy scenarios. When *Speed* is more important, the RTAB-Map showed the best score with 0.96. In a case where *Autonomy* has a higher priority, Gmapping also showed a comparable performance of 0.92 with the DNN-based BC.

## 1. Introduction

Robots play a pivotal role in enhancing human lives by undertaking tasks that are rigorous or hazardous. Recent advances in robotics and AI have revolutionized various sectors, including business, society, and personal spheres. Beyond their precision and consistency, robots exhibit adaptability to diverse environments, eliminating dangerous jobs in hazardous conditions. Capable of lifting heavy loads, handling toxic substances, and performing repetitive tasks, robots contribute to accident prevention, saving both time and money for companies. Robots also bring a lot of benefits to the agricultural sector, which makes good-quality food products feasible for the growing population. From precision planting and crop monitoring to weed detection and autonomous harvesting, robots offer a spectrum of applications [1]. No matter which application of robots in the agricultural field is considered, in most cases, navigation in the agricultural environment is needed. Despite the importance of robot navigation in agriculture, the number of articles in this field is considerably low. Autonomous navigation in the outdoor agriculture environment is relatively trickier than in the conventional urban environment, such as industries and indoor settings. Due to the dense nature of the agricultural field and uneven surface, it is difficult to map the environment for navigation using the traditional Simultaneous Localization and Mapping (SLAM) algorithms [2]. Also, the outdoor environment brings new challenges due to different lighting and weather conditions, which affect mostly vision-based mapping and navigation. In this research, we conducted a comprehensive comparative analysis of different navigation methods, including SLAM algorithms and Deep Neural Network (DNN)-based Behavior Cloning (BC) in an outdoor agriculture environment. SLAM algorithms are widely categorized into laser-based, vision-based, and multi-sensor fusion algorithms. Laser-based algorithms use the LiDAR sensor for mapping and localization. Vision-based algorithms use the camera, and fusion-based algorithms use both the camera and LiDAR for mapping and localization. For fair comparison, we use algorithms from laser-based (Gmapping [3], Cartographer [4]) and vision-based (RTAB-Map [5]) algorithms for this comparative study. We compare these SLAM algorithms with DNN-based BC [6] navigation, which is a map-free approach. This exploration aims to provide insights into the effectiveness and applicability of these methods in enhancing the control and navigation capabilities of robots in the agricultural landscape. The comparative assessment of vision-based, laser-based, and DNN-based BC algorithms is grounded in their navigation performance in a simulated orchard farm environment. Performance metrics derived from [7], encompassing driving deviation, completion time, success rate, and human intervention, constitute the basis for this comprehensive evaluation. The results show that DNN-based BC algorithms have promising capabilities for navigation in the agricultural environment. Table 1 shows the summarization of the navigation techniques along with the sensors that are analyzed in this paper.

So far, to the best of our knowledge, there is no concrete comparative analysis study of laser-based and vision-based SLAM algorithms and DNN-based BC approach specifically for the agricultural environment. In this paper, we compare three different mapping algorithms: two laser-based and one vision-based SLAM algorithms, which are GMapping, Cartographer, and RTAB-Map, respectively. We test the navigation using Adaptive Monte Carlo Localization (AMCL) for laser-based techniques, and RTAB-Map uses its own localization mechanism for vision-based navigation. DNN-based BC uses a DNN model trained by an expert’s demonstration to control the navigation of the robot.

The major contributions of this work are as follows:

- A new comparative study for autonomous navigation including the behavior cloning method is proposed.
- We tested popular SLAM algorithms developed for indoor environments in the agricultural outdoor environment and validated their performance.
- So far, to the best of the author’s knowledge, there is no comparative analyses of SLAM algorithms with DNN-based BC techniques together.

## 2. Related Work

We conduct our literature review into two broad categories, namely SLAM comparative study and DNN-based BC. A detailed review of these techniques is explained below.

### 2.1. SLAM Comparative Study

SLAM algorithms are broadly categorized into three main categories based on the type of sensor modalities they incorporate, namely: (1) laser-based SLAM algorithm, (2) vision-based SLAM algorithm, and (3) multi-sensor fusion SLAM algorithm. Laser-based SLAM generally uses single or multiple-line LiDAR sensors, which are referred to as raw-range scan sensors. Vision-based SLAM algorithms use cameras like monocular, binocular, or RGB-D to extract the features, commonly referred to as feature-based SLAM techniques. The multi-sensor fusion SLAM algorithm uses both laser and camera sensor modalities.

The agricultural environment is more challenging for mapping and localization than the traditional indoor and industrial environments because agricultural environments experience different weather, lightning conditions, and seasonal changes, which can pose difficulties for SLAM algorithms designed for urban or industrial environments. Dynamic growing crops can make it challenging to reconstruct the 3D structure of agricultural environments. Also, achieving a globally consistent map is challenging in environments where any mobile robot’s self-localization can suffer from imprecision. Therefore, consistent alignment of multiple 3D sub-maps when generating a global 3D map is a significant challenge [8]. These challenges highlight the unique complexities of agricultural environments and the need for specialized approaches to address them within the context of SLAM algorithms.

The specific parameters utilized by Kasar, A. et al. compare the performance of two popular visual SLAM algorithms, RGBD-SLAM and RTAB-Map, Ref. [9] on the TUM RGBD Dataset [10]. They evaluated the performances using Absolute Trajectory Error (ATE) and Relative Pose Error (RPE). The ATE measures the difference between the estimated trajectory and the ground truth trajectory, while the RPE measures the difference between relative poses estimated by the algorithm and the ground truth relative poses. These parameters were used to evaluate the accuracy and robustness of the algorithms in different situations and camera motions.

Garigipati et al. conducted an evaluation of eight prominent open-source 3D LiDAR and visual SLAM algorithms [11]. These algorithms were chosen based on their status as state of the art and on their widespread usage, with implementations in Robot Operating System (ROS) [12]. The selected algorithms encompass both laser-based and visual methods, with four algorithms utilizing LiDAR data and four employing visual data. Additionally, the algorithms include both full SLAM (Simultaneous Localization and Mapping) systems and odometry algorithms. The primary distinction between odometry and full SLAM algorithms lies in their approach to estimation and optimization. Odometry algorithms typically perform incremental estimation on a frame-by-frame basis, occasionally incorporating windowed local optimization techniques. In contrast, full SLAM methodologies aim to maintain global consistency by integrating loop-closure detection mechanisms. These mechanisms are utilized to identify previously visited locations, enabling corrections to pose estimation errors and ensuring overall global consistency within the map. The evaluation criteria used in the paper include the following: (a) Relative Pose Error (RPE) and Absolute Pose Error (APE). These metrics were used to assess the accuracy of the algorithms in estimating the robot’s pose in both indoor and outdoor environments. (b) Loop-closure capability: The ability of the algorithms to detect loop closures and maintain global consistency in the estimates. (c) Drift from the starting point: This metric was used to compare the quality of the SLAM estimates in a stationary robot scenario with moving objects in the view. (d) Computational resources: The paper provides a comparison of the required CPU resources for running the different algorithms, including average and peak CPU usage. These criteria were used to systematically analyze and compare the performance of the selected SLAM implementations in various scenarios, such as the mounting position of the sensors, terrain type, vibration effect, and variation in linear and angular speed of the sensors.

Liu, X. et al. compared three kinds of laser SLAM algorithms: Hector SLAM, Gmapping, and Cartographer. These algorithms were chosen because they are widely used in the field of autonomous robots and are effective in indoor mapping experiments [13]. The authors aimed to compare the accuracy and efficiency of these algorithms in a real environment experiment. The authors used several evaluation metrics to compare the performance of the three algorithms. These metrics include the length of the indoor features measured by traditional methods, the drawing length of the features obtained by each algorithm, the maximum difference, the minimum difference, and the medium error of the graphing results of the three algorithms. The authors also analyzed the cumulative error caused by each algorithm.

Li, Z. et al. used Gmapping SLAM, Hector SLAM, Cartographer SLAM, and ORB SLAM and compared their advantages and disadvantages in a complex indoor environment [14]. GMapping and Hector SLAM are laser-based SLAM techniques whose performances are evaluated against the ORB SLAM, a vision-based method. The evaluation criteria are an algorithm efficiency comparison in terms of time, a comparison of construction effect on different algorithms with dynamically changing environments, and mapping accuracy.

Ibragimov, I.Z. et al. performed a comparative study on four vision-based algorithms, namely ORB-SLAM, Monocular DPPTAM, Stereo ZedFu, RTAB-Map, and evaluated the performance with state-of-the-art Laser-based algorithm Hector SLAM [15]. The algorithm uses different sensor modalities like conventional cameras, stereo or depth cameras (ZED stereo camera and Kinetic depth sensor), and LiDAR. The evaluation metrics are maps analysis and odometry comparative analysis.

Yu, J. et al. compared the cartographer algorithm with Gmapping and Hector SLAM [16]. The comparison was conducted based on three factors: synchronous positioning and mapping accuracy, computational complexity, and mapping efficiency. They computed absolute value data and absolute error data. Finally, they concluded that Cartographer is better for indoor environments. They considered two scenarios: simulation and systems in actual experiments, which were carried out to evaluate the suggested approach.

Tiozzo et al. (citation missing) conducted a comparison of four Laser-SLAM algorithms, focusing on their ability to reconstruct point clouds of surveyed environments and their computational requirements [17]. The algorithms evaluated were Real-Time Appearance-Based Mapping (RTAB-Map), Lightweight and Ground Optimized LiDAR Odometry and Mapping (LeGO-LOAM), Direct LiDAR Odometry (DLO), and hdl-graph-slam. These algorithms were selected for comparison as they exclusively utilize LiDAR data without relying on additional sources such as Inertial Measurement Systems (IMUs), encoders, or Global Navigation Satellite Systems (GNSS). They offer different approaches to downsampling and scan matching, leading to variations in the quality of the final point cloud reconstruction. Additionally, each algorithm employs distinct data structures for storing and accessing information during the mapping process, impacting computational efficiency. Furthermore, these algorithms are particularly suitable for embedded systems and mobile robotics applications due to their lightweight nature and efficient performance.

Khan, M.S.A. et al. used Agribot to do SLAM in a greenhouse environment that is considered an indoor place [18]. They mainly focused on four main things: (a) RTAB-Map using RGBD-SLAM algorithms, (b) Hector SLAM, (c) 2D map generation, and (d) handling the computation cost. They compared the total number of frames, loop closure detection, localization (based on accuracy), and mapping (based on accuracy).

Ratul, M.T.A. et al. implemented Gmapping in ROS. What they did was design four different agricultural environments, and then they compared the map made for these four environments and the time needed for map building [19]. And finally, Habibie, N. et al. used the same robot as we used in their environment [20]. They tried to adjust the SLAM Gmapping for outdoor use and then compared Gmapping and Hector-SLAM In ROS, and they compared the maps that were built with them.

### 2.2. Behavior Cloning

Pomerleau, D et al. pioneered End-to-End (E2E) learning, categorized as DNN-based BC for steering a car on a road, using a fully connected neural network with inputs from a camera and a laser range finder [21]. They implemented a shallow neural network.

DAVE (DARPA Autonomous Vehicle) adopted a similar E2E approach for an off-road radio control car [22]. It eventually learned to extract and process information from raw video input, including 3D image extraction, edge detection, object detection, and obstacle avoidance. They used an RC truck for training instead of a simulator.

Chen, Z. et al. introduced DAVE-2, which contributed to the rise in popularity of behavioral cloning with E2E learning [23]. It is primarily influenced by ALVINN and utilizes a Convolutional Neural Network (CNN) to extract visual information from front camera photos. They introduced PilotNet to scale up deployment.

Bojarski et al. demonstrated the application of deep neural networks and E2E learning for vehicle control [24]. They offered a clear explanation of E2E learning without introducing novel concepts.

Wang et al. proposed an angle-branched network technique for E2E learning. It improved steering angle and throttle predictions by incorporating sub-goal angle predictions [25]. They found that including the sub-goal angle enhanced the driving model’s performance.

Wu et al. presented an E2E driving model based on a Convolutional Long Short-Term Memory (Conv-LSTM) neural network [26]. They included a Multi-scale Spatiotemporal Integration (MSI) module for encoding diverse spatiotemporal input for steering angle prediction. It utilized future sequential information in model training to enhance spatiotemporal characteristics. They evaluated performance using public Udacity data and a real-time autonomous vehicle, noting the need for improved steering control and visualization.

## 3. Methods

### 3.1. SLAM Algorithms

Based on our comprehensive literature analyses, we select three different mapping algorithms, including two laser-based algorithms and one vision-based algorithm. For the laser-based algorithm, we select Gmapping and Cartographer, and for the vision-based algorithm, we select the RTAB-Map. The environment that we use for our work is an orchard farm with uneven terrain, which makes both mapping and navigation challenging. The challenges that we came across are discussed in the Section 6.

To navigate an environment with a SLAM algorithm, a robot needs to explore it to make an occupancy map [27].

#### 3.1.1. Laser-Based Mapping

##### Gmapping

This method uses two strategies to improve Rao-Blackwellized Particle Filters (RBPFs) in addressing Simultaneous Localization and Mapping (SLAM) challenges with grid maps. Firstly, a sensor-considerate proposal distribution is introduced, enhancing particle accuracy and reducing the need for numerous particles. Secondly, an adaptive resampling technique ensures particle diversity, minimizing the risk of depletion. Together, these methods enhance RBPFs’ precision and efficiency in SLAM applications with grid maps. Gmapping is one of the most popular mapping algorithms used in ROS, and it is mostly used as a base to compare the results.

##### Cartographer

Google’s Cartographer presents a dynamic indoor mapping solution utilizing a sensor-equipped backpack to create 2D grid maps at a 5 cm resolution. Laser scans seamlessly integrate into sub-maps, and consistent pose optimization, opting for it over a particle filter, tackles error accumulation. Completed sub-maps play a crucial role in loop closure during scan matching, guaranteeing swift closure of loops when revisiting locations. Optimizing every few seconds adheres to a soft real-time constraint, employing a branch-and-bound approach and precomputed grids for each finished submap, ensuring efficient performance with modest hardware requirements.

#### 3.1.2. Vision-Based Mapping

##### RTAB-Map

RTAB-Map, integrated into ROS since 2013, is a graph-based SLAM approach with an external odometry input. It employs a graph structure with nodes and links, where the Short-Term Memory (STM) module creates nodes containing odometry pose, raw sensor data, and additional information. Links include Neighbor, Loop Closure, and Proximity links, serving as constraints for graph optimization. Graph optimization decreases odometry drift, and outputs like OctoMap and Point Cloud are assembled and published. RTAB-Map’s memory management is divided into Working Memory (WM) and Long-Term Memory (LTM), limiting graph size for real-time constraints. A weighting mechanism prioritizes important locations, transferring nodes from WM to LTM based on thresholds and heuristics. Loop closures with WM locations can bring back neighbor nodes from LTM for further processing. This approach allows incremental map extension and localization in previously visited areas as the robot moves.

Loop Closure is a sub-algorithm of SLAM that decides if the robot has previously visited the same place or not [28]. It helps the SLAM algorithm optimize the accumulated errors and the robot’s pose estimation. It is essential yet hard to implement, especially in the orchard farm, where the different locations appear to be similar to each other, forcing the loop closure even when it is not needed.

RTAB-Map uses different graph optimization strategies; the one we used for this research to avoid the unnecessary loop closure issue is TORO-based graph optimization [29]. The TORO (Tree-based netwOrk RObust optimization) algorithm is a graph optimization technique commonly used in the field of robotics, particularly for simultaneous localization and mapping (SLAM) tasks. TORO is designed to optimize the pose graph, representing the relationships between robot poses and observed features in a 2D or 3D space. The goal is to refine the estimated poses and landmarks to improve the overall accuracy of the robot’s trajectory and map.

After building a map, localization algorithms are used to identify a robust pose in navigation. Laser-based localization uses Adaptive Monte Carlo Localization, and the RTAB-Map uses the RTAB-Map navigation stack for localization. These localization techniques are described in the next section.

#### 3.1.3. Laser-Based Localization

##### Adaptive Monte Carlo Localization

Adaptive Monte Carlo Localization is based on a probabilistic model using particles for the robot’s 2D pose estimation using sensor data [30]. It uses the prior map generated by SLAM mapping and compares it to the real-time sensor data to determine the true 2D pose of the robot. AMCL is widely used in different robot applications due to its low computational power, and it is easy to deploy in a real-time environment.

#### 3.1.4. Vision-Based Localization

##### RTAB-Map Localization

RTAB-Map uses its own navigation stack in ROS for localization. It uses the previously generated map and the real-time camera data to help the robot navigate through the environment and estimate its true pose dynamically.

For navigation, we used AMCL (Adaptive Monte Carlo Localization) for both laser-based algorithms (GMapping and Cartographer), while RTAB-Map employed its own navigation stack. Our evaluation involved comparing the navigation outcomes of AMCL and RTAB-Map algorithms, utilizing the maps generated by Gmapping, Cartographer, and RTAB-Map. In addition, we compared these results with the DNN-based BC, a map-free navigation approach. This method relies on expert navigation data, specifically images and steering information, to emulate navigation behavior within the environment.

### 3.2. Behavior Cloning

In DNN-based BC, a DNN model is trained to replicate an expert’s behavior, with the expert being a human driver in the context of autonomous driving. The model is optimized to emulate driving commands, including steering, acceleration, and braking, based on sensory input recorded while the human drove. The simplicity of gathering extensive human driving data makes it effective for straightforward tasks like lane following.

#### 3.2.1. Data Collection

The data for the DNN-based BC can be defined as:

(1) $$D^{n}={\left\{o^{n}\left(i\right),a^{n}\left(i\right)\right\}}_{t=1}^{T},$$

where $o^{n}\left(i\right)$ is the observation collected at the time *i*, and $a^{n}\left(i\right)$ is the corresponding actions (i.e., steering, throttle, brake) at that particular time, *T* represents the total time step of the data collection.

The robot generated several topics in ROS, out of which three were publishing the data that were of interest for data collection. The vehicle control topic published information about steering angle, throttle, and brake. The base pose topic published information regarding the position, angular, and linear velocity of the robot, and the camera image topic published the images it captured at the particular time instance.

**Figure 1:**
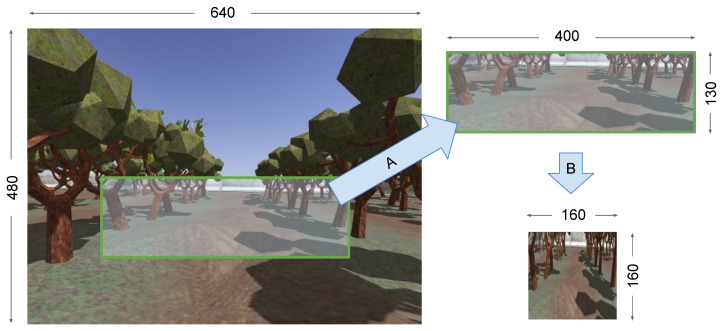
The camera captures images in the size of 640 × 480 during the data collection phase of DNN-based BC. The robot roams around the orchard farm and captures these images using a camera along with the respective steering angle, throttle, brake, timestamp, and robot pose. The region (120, 240)–(520, 370) is cropped (A) and resized to 160 × 160 (B) to feed in the DNN-based BC for training. The original 640 × 480 contains irrelevant or extra information that might not be useful for training the network.

Figure 1 shows the original image collected during the data collection and the cropped image generated for the training of DNN-based BC, respectively.

#### 3.2.2. Neural Network

**Figure 2:**
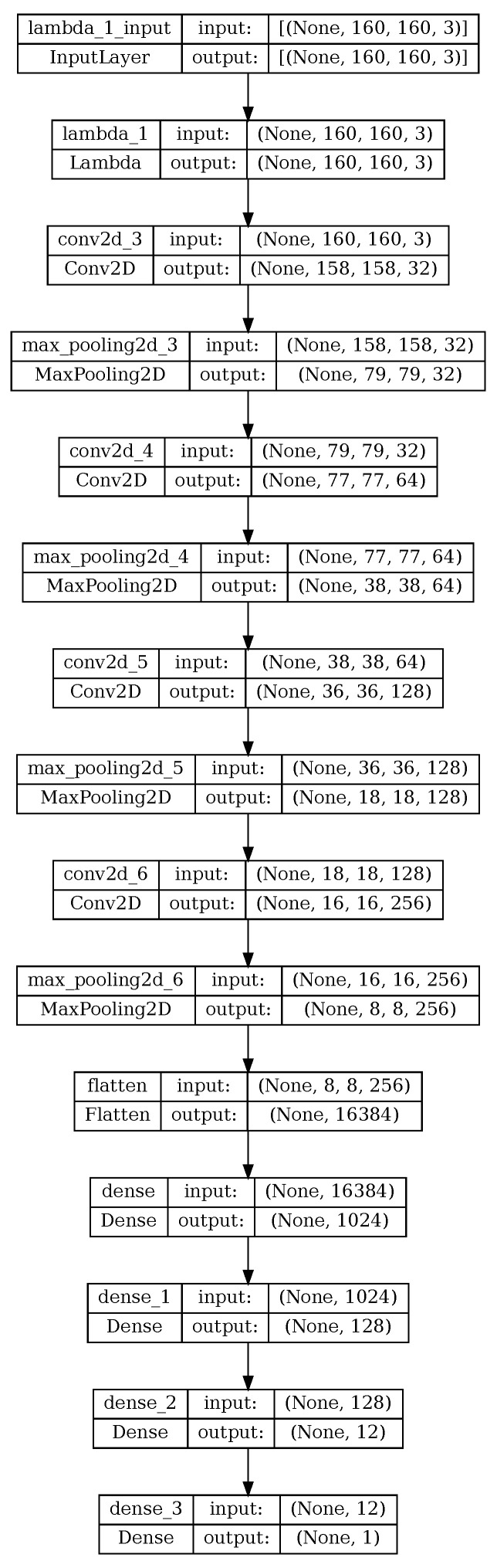
Neural Network Architecture for DNN-based BC. It comprises a lambda layer that is responsible for the normalization of the data, followed by four convolutional layers with the max-pooling layers. The convolution layers are responsible for extracting the important features from the image. The convolution layers are equipped with filters of sizes 32, 64, 128, and 256, respectively. The max pooling layer is responsible for downsampling. Subsequently, three dense layers and a flatten layer are employed to converge the downsampling into a single output, i.e., the steering angle.

The neural network architecture is inspired by the NVIDIA PioletNet [6] architecture. It comprises four convolutional layers, each followed by a corresponding max-pooling layer, along with an additional four dense layers. The initial layer, serving as a lambda layer, is dedicated to normalizing the input data. Conv2D layers are strategically employed to extract crucial features pertinent to navigation tasks, configured with filter sizes of 32, 64, 128, and 256, all featuring a kernel size of three. The Rectified Linear Unit (ReLU) activation function is uniformly applied across all layers in the network. MaxPooling2D layers facilitate downsampling. Ultimately, a flattened layer precedes three Dense layers that converge into a single output, representing the steering angle. The model’s architecture is visually presented in Figure 2. We used the Mean Squared Error (MSE) for the difference between the predicted steering angle and the ground truth as a loss function for our training. The reason for choosing MSE is that it puts more weight on larger errors, which could help faster training.

#### 3.2.3. Data Normalization

**Figure 3:**
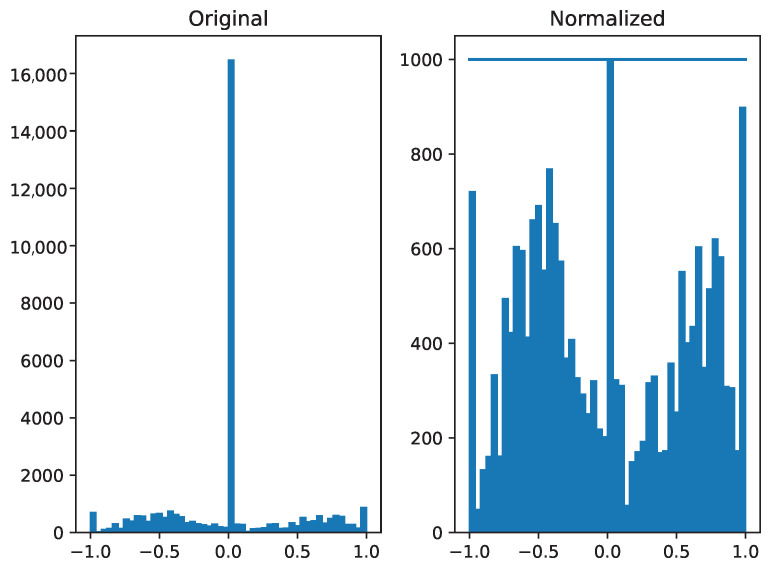
Representation of the distribution of steering angle between 0.0 to 1.0 in the data. The left figure illustrates the distribution of raw data collected during data collection, and the right figure demonstrates the distribution of the normalized data acquired during preprocessing. The raw data possesses more images with steering labeled as zero; therefore, it is necessary to normalize the data before training to avoid bias.

Data normalization is a preprocessing step for training. Here, for data normalization, we tried to make a balance between data available with different steering angle commands. As driving in a lane consists of many images with zero steering, we tried to remove some of the images that are near zero steering angle in order to balance the data. This is mainly because if we do not give the neural network balanced data, it would become biased to the specific steering and would not give the steering that is needed in different scenarios. Figure 3 demonstrates the distribution of steering angle in raw data as compared to the normalized data.

The 1000 on the y-axis suggests the maximum number of samples per steering angle. The raw data data before this normalization shows that the steering angle zero has 16,000 samples. In order to make this training data balanced to avoid bias, we perform normalization on the steering angle. As predicting the steering angle is the regression task, the aim is to predict a closer prediction value to the ground truth.

#### 3.2.4. Training

**Figure 4:**
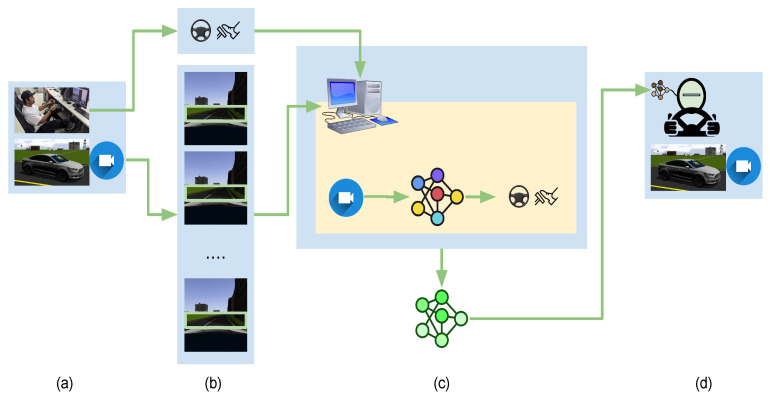
DNN-based BC: (**a**) A human driver drives a vehicle as we collect driving data. (**b**) The driving data, including the front camera images with synchronized control signals, are saved in storage. The collected data must have all the necessary features that can be expected in a testing phase of the neural network. (**c**) The training station is where a neural network is trained with the collected data to associate input with output. (**d**) the trained neural network is deployed to the DNN-based controller who drives the vehicle by using inferred steering angles, throttle, and brakes. Adapted from [7].

DNN-based BC learns from expert demonstrations. It can be defined using the following equation:

(2) $$o^{n}\to \mathrm{policy}:\pi_{\theta }^{n}\to a^{n},$$

where $o^{n}$ is the n number of observations to train a CNN and derive an optimal policy π, which later on predicts the future action of the robot. The overview of DNN-based BC can be illustrated in Figure 4.

#### 3.2.5. Performance Metrics

The metrics to define the performance of selected techniques are driving deviation, completion time, and human intervention. We derive these performance metrics from the Online Performance Evaluation Metrics Index (OPEMI) [7].

##### Driving Deviation

The driving deviation is defined as the standard deviation of the measured distance (Equation (3)) from the desired path to the robot’s current location while it is navigating.

(3) $$\mathrm{Distance}=\frac{|Ax_{0}+By_{0}+C|}{\sqrt{A^{2}+B^{2}}},$$

where ($x_{0}$, $y_{0}$) is the robot location, and *A*, *B*, and *C* are the coefficients of a straight line represented by Ax+By+C=0.

##### Completion Time

Completion time is the average navigation time for a robot to reach the target from the starting point in lanes. The time when humans intervene to help the robot is included in the completion time. Therefore, the completion time is not additionally penalized for human interventions.

##### Autonomy

Autonomy refers to the ability of the robot to reach its target without any human help. Sometimes, human intervention was required if the robot got stuck in the trees or puddled in the ground. It can be calculated by Equation (4), which is inspired by autonomy metric [6], where the number of interventions is the number of times humans intervene to help the robot, and elapsed time is the total time the robot navigates from the starting point to the target. The original autonomy metric uses 6 s to penalize the human intervention. It took around 15 s for a human to restore the pose of the robot on the orchard farm. Thus, we modified the penalization to 15 s instead of 6 s.

(4) $$\mathrm{Autonomy}=\left(1-\frac{N\times 15\;(s)}{T(s)}\right)\times 100,$$

where *N* is the number of human interventions, and *T* is the elapsed time.

## 4. Experimental Setup

We performed our experiments using three different mapping algorithms and DNN-based BC. For vision-based, we use RTAB-Map and its own navigation to validate the results. We used Gmapping and Cartographer for laser-based techniques and used the AMCL algorithm for navigation. For DNN-based BC, we derived inspiration from the PilotNet [6]. We tried to navigate the robot in five different lanes and measured the performance using the metrics defined in Section 3.2.5.

### 4.1. Environment

The simulation environment comprises two important components: the robot and the agricultural environment. We used AgileX Robotics’ Scout 2.0 [31] robot. For the simulation of the agricultural environment, we used an orchard farm [32]. A detailed description of these components is described below.

#### 4.1.1. Scout 2.0

**Figure 5:**
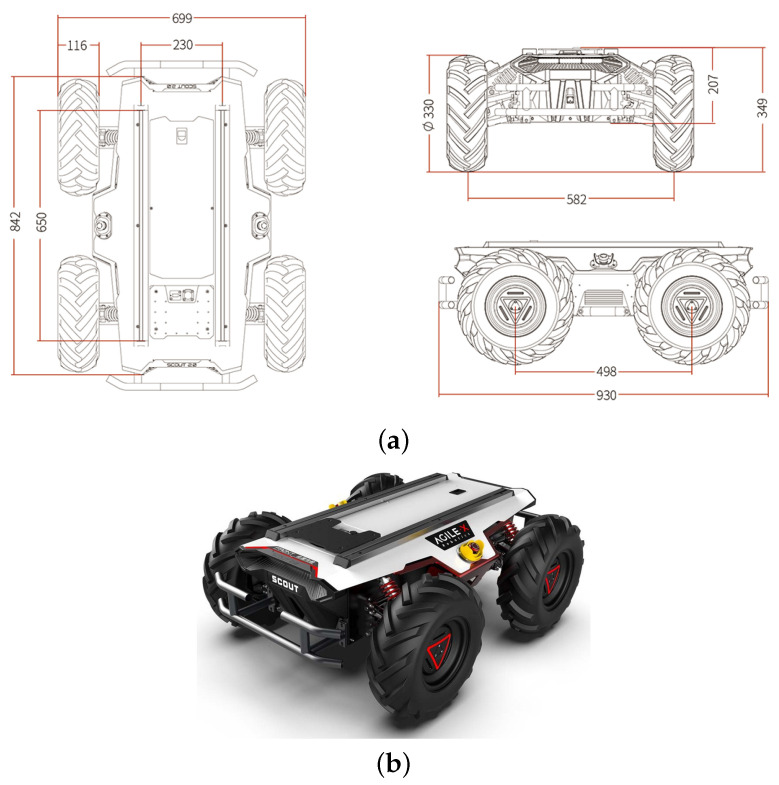
(**a**) The dimensions of Scout 2.0 and (**b**) The side view of Scout 2.0.

Scout 2.0 is a mobile robot that features low energy consumption, an extended battery life, a robust yet compact frame, and adaptable software interfaces. Its drive type is four-wheel differential drive. Each wheel has a 400 W brushless servo motor. The dimension of Scout 2.0 is 930 mm × 699 mm × 349 mm (length, width, and height) with weight of 68 kg. More importantly, it is well-equipped to tackle a variety of challenges and can work well in the agricultural environment. It can facilitate the effortless installation of modular hardware packages and sensors. Also, it provides an open-source communication standard and software packages based on ROS, hence serving our purpose better for this research. Figure 5 shows the dimensions and the Scout 2.0 side view.

**Figure 6:**
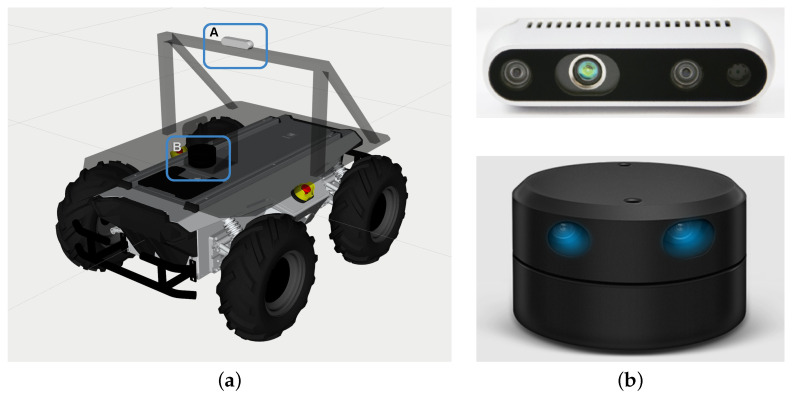
(**a**) Placement of the Intel RealSense camera and LiDAR on Scout. The “A” shows the position on the frame the camera is mounted on. The camera is deliberately placed higher to avoid capturing part of Scout’s body in the frame of the camera. The “B” is the position where the 2D LiDAR is mounted to create a 2D point cloud. It is strategically pointed slightly ahead of the body of the Scout to avoid generating a point cloud for the body of the Scout. (**b**) Intel D435i RealSense depth camera (**top**) and YDLiDAR G4, 2D LiDAR (**bottom**).

We added a frame to the Scout 2.0 to mount a 2D LiDAR, and a camera. We used a simulated YDLiDAR G4 [33] and Intel D435i RealSense depth camera [34]. Figure 6 shows the placement of the camera and lidar and camera on the Scout 2.0 in the simulator.

#### 4.1.2. Orchard Farm

**Figure 7:**
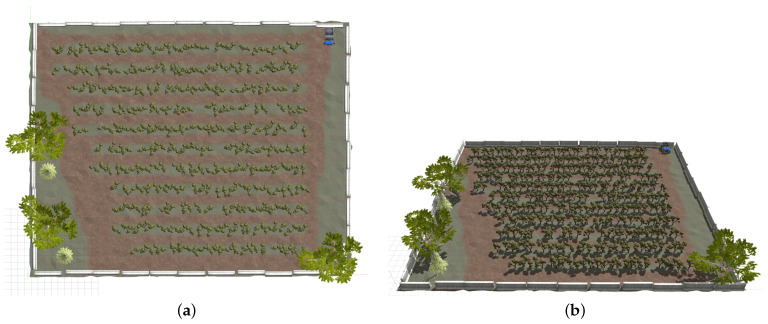
(**a**) The bird-eye view of the orchard farm in the Gazebo simulator. It contains 12 lanes where the robot should be able to navigate. The barriers are added to the surroundings of the farm as an added obstacle. Three oak trees and a vehicle prop in the top right corner are also added as obstacles. (**b**) Orchard farm profile.

The original orchard farm is a simulated world provided by Clearpath Robotics [32]. It is mainly used for the simulation of agricultural settings for different robotics applications. We modified it by adding barriers around the field, three oak trees, and a parked vehicle. The height of the orchard farm is 38 m, and the width is 47 m. Figure 7 shows the orchard farm we used for this research.

### 4.2. Simulation Environment

**Figure 8:**
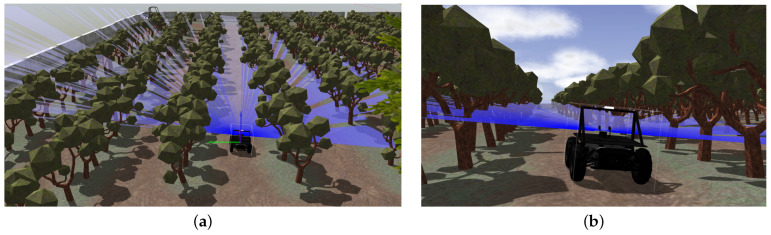
Scout 2.0 in action. The blue rays are a visualization of a simulated LiDAR beam. (**a**) top view of how the simulation environment looks like when Scout 2.0 is navigation or driving in the orchard farm. (**b**) Closer look of the Scout 2.0 in the orchard farm while driving/navigating. The camera and LiDAR can be seen mounted on the Scout 2.0.

We used the ROS to simulate our robot and the orchard farm environment. We made some changes in the orchard to make it accustomed to our needs for this research. We used the Gazebo Simulator [35] integrated with ROS for this purpose. Figure 8 shows the Scout 2.0 in action in the Gazebo simulator in the orchard farm.

**Figure 9:**
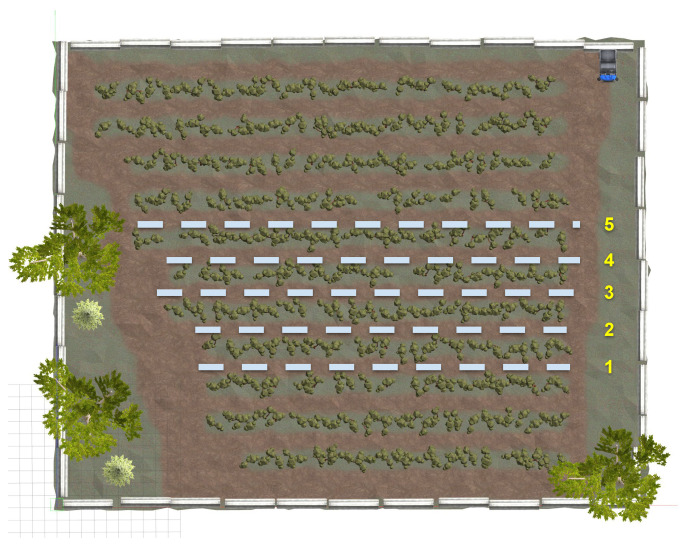
Orchard farm with lanes chosen for the navigation testing of the SLAM algorithms and DNN-based BC. The Scout 2.0 should be able to self-navigate from start to end in these lanes labeled as 1, 2, 3, 4, and 5.

Figure 9 illustrates the orchard farm in the Gazebo simulator we have used for our experiments. It consists of twelve aisles surrounded by rows of trees. We referred to these aisles as lanes where the robot should be able to navigate by itself. The distance between the trees varied throughout the farm. We tested our approaches by navigating the robot in five different lanes (from lane 1 to lane 5), illustrated with numbers in Figure 9. For better evaluation, we tested each lane for navigation twice. First, we tried to move the robot to the end of the lane from its initial position (we assumed that point to be the starting point of that lane). Once the robot reached its target (that is, the end of the lane), we instructed the robot to navigate to its initial position in the lane. We refer to this as the left-to-right and right-to-left navigation for the respective lane. We then evaluated their performances based on driving deviation, completion time, and human intervention.

### 4.3. Implementation

#### 4.3.1. Agricultural Robot

**Figure 10:**
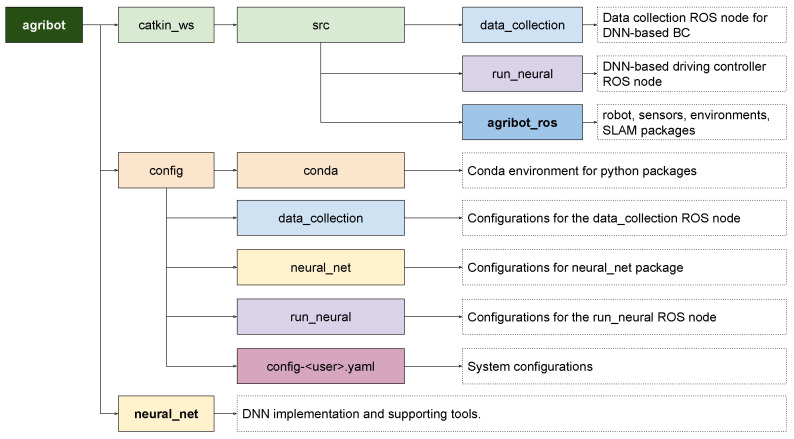
Software system overview. The agribot provides a framework for DNN-based BC, including data collection for BC, training and testing DNNs, heatmap visualization, etc. The agribot_ros is a ROS package for supporting LiDAR and vision-based mapping and navigation of SLAM algorithms.

We designed and implemented a DNN-based BC framework for an agricultural robot: agribot [36]. The agribot supports data collection for BC, training and testing DNNs, visualization of the heatmap, and more. We also implemented an ROS package, agribot_ros to support LiDAR-based and vision-based mapping and navigation of various SLAM algorithms, including Gmapping, Cartographer, and RTAB-Map [37]. The overview of the agribot and agribot_ros is depicted in Figure 10.

**Figure 11:**
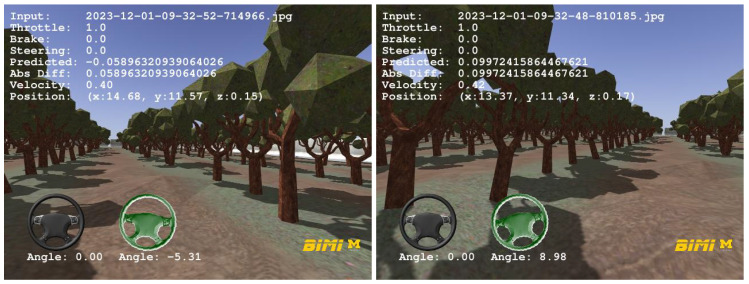
Visual comparison between ground truth and predictions. https://www.youtube.com/watch?v=FeGPh5DjoWg (accessed on 2 April 2024).

To qualitatively evaluate a trained DNN, agribot provides a comparison between ground truth and predictions of control signals (Figure 11).

#### 4.3.2. SLAM Algorithms

For Gmapping, we used the 2D LiDAR scanner to map the environment. Mapping the environment using Gmapping was pretty straightforward and easier as compared to the other SLAM algorithms. We used the Inertial Measurement Unit (IMU) data for the Cartographer along with the 2D LiDAR because of the drifting issues it caused during mapping. We tried to map it without an IMU sensor; however, due to the uneven terrain, the odometry of the robot drifts from the plane at a certain time during the mapping. We often came across the issue of loop closure during the mapping. As RTAB-Map is the only vision-based algorithm we used for this research, it made us realize that vision-based SLAM algorithms are more prone to loop closure problems. We overcome this issue by using a TORO-based graph optimization algorithm. The RTAB-Map uses the RealSense camera to map the environment.

Apart from these minor changes, the general configuration of the environment and robots were the same for the purpose of generality.

#### 4.3.3. DNN-Based BC

For DNN-based BC training, the images, along with their respective labels (steering angle, throttle, brake, robot’s pose, and timestamp), were collected using the RealSense camera in the orchard farm environment. Our purpose was to collect efficiently and to collect enough data to train the model so that the robot could pass through the lane without hitting any obstacles (trees in our case) without any human intervention. Also, in the data collection phase, the challenge was the surface of the farm, which was not flat terrain. Since it was an agricultural environment, the path was not smooth, and there were a lot of uneven potholes. For that reason, our purpose was to make the robot familiar enough with the environment to drive through these uneven surfaces in the testing phase. In order to fulfill that requirement, we deliberately steered slightly every now and then during our data collection.

We collected around 45,000 images along with their respective steering angle, throttle, brake, position, velocity, and angular velocity. The total size of the dataset was around 4 GB, and the images were 640 × 480 pixels in size. The next process was data cleaning. As we were only interested in autonomously driving the robot in the lane, we eliminated the front and back corridor images of the farm in the data cleaning. Furthermore, we also eliminated the parts where the vehicle was making a sharp turn, mainly during the transition from one lane to another.

In Figure 3, a visual representation depicts the data both before and after normalization. A total of 30% of the training data was allocated for validation purposes. The model underwent training for 15 epochs out of the maximum 100 epochs. The early stopping patience was set to 3. We used a batch size of 32 for training. The input data were augmented by flipping and brightness adjustments (randomly selected between 5% darker and 15% brighter) to enhance the robustness of the model. Additionally, for effective training, images were cropped (160 × 160 × 3, Figure 1) before it was fed to the neural network, focusing on the most relevant portions for the driving task, thereby facilitating accelerated and improved learning for the model. We used TensorFlow 2.15 with Keras framework 2.14.0. Python 3.9 was used, along with ROS Noetic distribution in Ubuntu 20.04. Python 3.10 was not compatible with ROS Noetic at the time we were performing the experiments. The hardware configurations comprised an NVIDIA GeForce RTX 2080 GPU of 8 GB RAM, with 32 GB memory, and Intel Core i9-9900 CPU.

**Figure 12:**
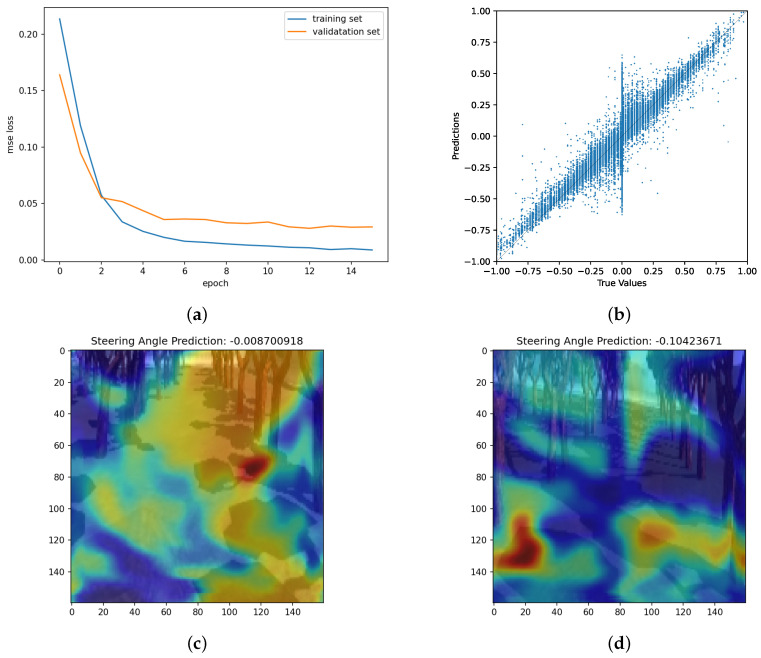
Training a DNN for BC. (**a**) Training and Validation Loss over the Number of Epochs. Originally, the number of epochs was set to 100, but it got early, stopping at the 15th epoch because the validation was not improving beyond around 0.048 for consecutive epochs. The early stopping patience is set to 3. (**b**) Scatter plot of the predictions and the true values. The nearer the values are to the straight line, the more accurate the predictions are from the DNN-based BC. (**c**,**d**) Examples of the activation heat map. The activation is overlapped on a cropped image that was fed to the DNN.

Figure 12a illustrates the performance graph of our training and validation loss over the number of epochs. The loss decreased in the past 15 epochs, and it got early stopping. Figure 12a also illustrates how accurate the prediction is used in the scattered plot. Also, how the quality of predictions improved over the time period during the training. Figure 12b shows how close the predicted values of the steering angles are to the ground truth. The closer the data point to the straight line in the center, the more accurate the predicted values are. The model is able to predict the values quite closely to the ground truth.

The reddish areas in Figure 12c indicate the steering angle value, −0.0087, which is a near zero value, meaning going straight, has been determined based on the road area ahead. Note that steering angle ranges are from −1 to 1 (right to left). In Figure 12d, the dark red area at the bottom left was the major reason for the steering angle, −0.104, which means turning the steering angle to the right because the image under the heatmap shows the robot is facing slightly left side with respect to the road area.

## 5. Results

We evaluated the performance of each model based on the driving deviation, completion time, and number of human interventions. We also navigate the robot twice in the same lane from left to right (LR) and right to left (RL) to evaluate the performances of selected approaches.

**Figure 13:**
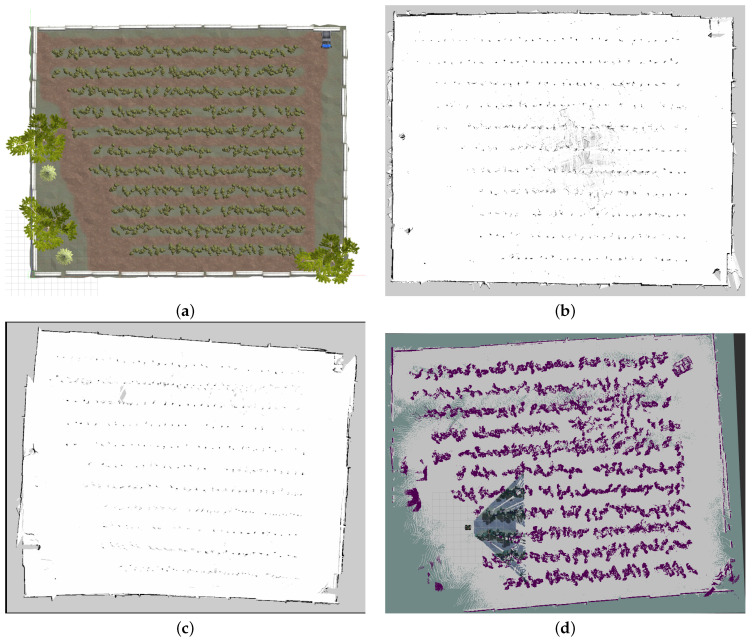
Orchard farm and maps built by different algorithms: (**a**) The bird-eye view of the orchard farm, (**b**) Map built by Gmapping, (**c**) Map built by Cartographer, and (**d**) Map built by RTAB-Map.

The maps generated by the Cartographer are slightly drifted. We suspect that the issue is because of the odometry of the robot, which uses the IMU sensor, and it does not align well with uneven terrains, and the map gets drifter. There are noises in some lanes in the map generated by RTAB-Map because of uneven terrain where the camera was not able to make the map correctly. The map quality can be evaluated in Figure 13. The map generated by RTAB-Map is a bit downgraded. We ran into odometry problems while generating the map for RTAB-Map using a camera. The map quality of GMapping was better among them all.

**Figure 14:**
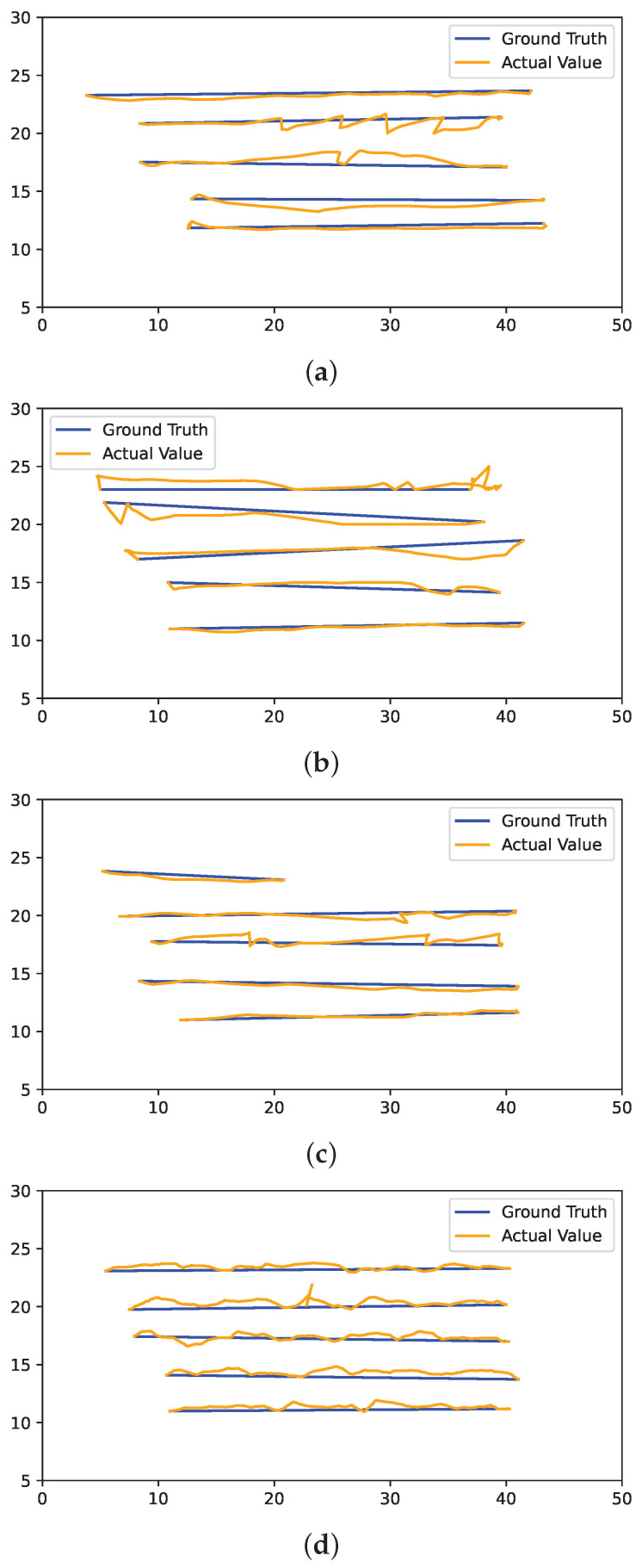
Driving deviations (left to right: LR) of (**a**) GMapping, (**b**) Cartographer, (**c**) RTAB-Map, and (**d**) DNN-based BC.

**Figure 15:**
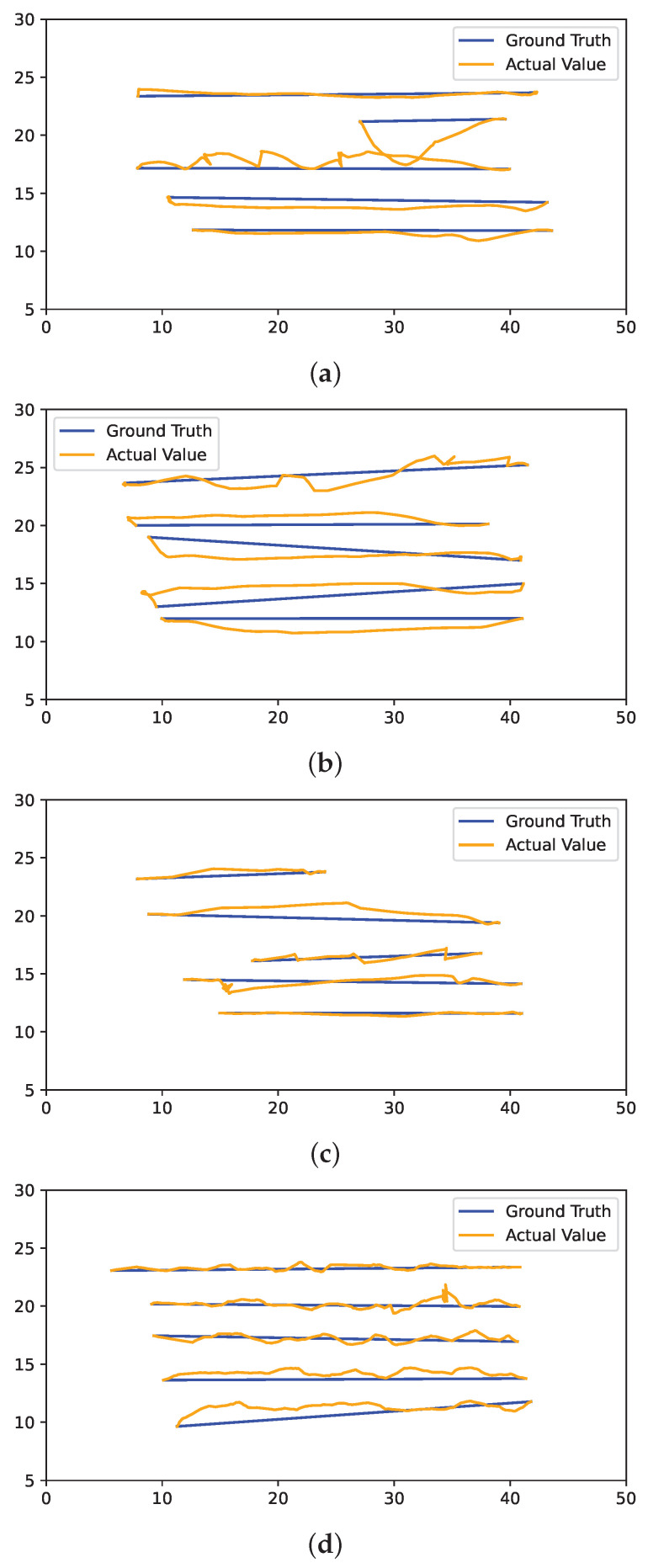
Driving deviations (right to left: RL) of (**a**) GMapping, (**b**) Cartographer, (**c**) RTAB-Map, and (**d**) DNN-based BC.

The driving deviation is not significantly improved or downgraded for any of the particular techniques. However, navigation based on the Cartographer-based mapping shows slightly more deviated driving than the other techniques. As the map generated by the RTAB-Map is noisy in some lanes, the robot was not able to navigate through some lanes, resulting in failure to achieve the navigation goal. Figure 14 and Figure 15 illustrate the driving deviation from a straight line in each selected lane for GMapping, Cartographer, RTAB-Map, and DNN-based BC from LR and RL navigation respectively. The robot was not able to move pass a certain point in the RTAB-Map driving deviation graph (Lane 5) because it considered the noise to be an obstacle to navigating through it.

Table 2 illustrates the average driving deviation, average completion time, and the autonomy metric of both driving from left to right and right to left. The table also shows the standard deviation of the driving deviation and the completion time to validate the results consistencies.

Table 3 shows a scaled evaluation of the performance index for each technique. We performed a min–max normalization to scale the performance metrics for better comparison. For driving deviation, we computed the standard deviation of the x-axis path the robot navigates through during testing. The completion time is measured in seconds. The table also shows the actual standard deviation of the driving deviation, average completion time, and average number of human interventions for each approach in LR and RL navigation.

For the sake of simplicity, we refer to the normalized driving deviation, normalized completion time, and autonomy metric as ($\tilde{DD}$), ($\tilde{CT}$), and ($\tilde{AT}$), respectively. Also, we considered the standard deviation of driving deviation, average completion time, and average number of human interventions as DD, CT, and AT, respectively.

We were able to autonomously navigate the robot using DNN-based BC. In contrast, we often needed to help the robot restore its pose and start navigating again. Also, DNN-based BC helps the robot to drive more precisely in a straight line as compared to the SLAM algorithms. However, we have deliberately scaled the throttle to drive slowly during the testing of DNN-based BC; therefore, the speed or completion time is compromised.

For simplicity, we have separately formulated the scaled evaluation of the performance index for each technique in Table 3.

## 6. Discussion

We realized that the agricultural environment is different from the conventional urban or industrial settings where most of the prior research work is done. We have come across issues such as uneven terrain, which often makes it difficult for the robot to move in the right direction (even with the right navigation commands). Not only does the robot need to make good navigational decisions, it should be able to compensate for the turbulence caused by uneven terrain. The uneven terrain also sometimes makes it difficult to map correctly with the selected SLAM techniques, which shows quite good performances in other environments.

Since we customized an orchard farm environment, it contains aisles surrounded by rows of trees. This makes each aisle similar to each other, making it difficult for the robot to distinguish between different aisles. This problem leads to loop closure issues during the mapping process, where the algorithm often tries to close the loop because it considers the unmapped positions as mapped before. We have particularly encountered loop closure issues while mapping with RTAB-Map (which is a vision-based technique). The observation made us realize that vision-based algorithms are more prone to loop closure issues when different locations in the environment are similar. To overcome the loop closure issue, we used the TORO-based Graph Optimization technique, which proved to be better than other optimization methods.

We have compared the performances of the SLAM algorithms and DNN-based BC using performance metrics discussed in Section 3.2.5. There can be different scenarios where one metric can be more considerable than the other. We have formulated these as the following equation:

(5) $$P=\omega_{DD}\left(1-\tilde{DD}\right)+\omega_{CT}\left(1-\tilde{CT}\right)+\omega_{AT}\left(AT/100\right),$$

where $\omega_{DD}$, $\omega_{CT}$, and $\omega_{AT}$ are variables to prioritize the precision driving (by using normalized driving deviation ($\tilde{DD}$)), faster completion time (by using normalized completion time ($\tilde{DD}$)), and autonomy (by using the autonomy metric AT) over one another in different scenarios. Note that $\omega_{DD}+\omega_{CT}+\omega_{AT}=1$. Weights can be determined based on use cases. Three sample scenarios are shown in the Table 4.

Compared to the SLAM algorithms, the DNN-based BC driving was pretty straightforward. Instead of mapping the whole environment, we collected the data as input images along with the respective steering angle to train a DNN. It was a lot less time-consuming than the mapping algorithms we used for this research.

## 7. Conclusions

In this research, we have comprehensively analyzed several SLAM algorithms and DNN-based BC navigation in the simulated outdoor agricultural environment. We further divided the comparative analysis of SLAM algorithms into laser-based and vision-based SLAM algorithms. We also propose an integrated performance indicator to compare one with another in different use cases. The DNN-based BC navigation technique seemed more accurate in navigation and less human intervention. If there is faster completion of navigation values, RTAB-Map can be a better choice. This integrated performance indicator can help determine which navigation technique must be selected in certain use cases. The algorithms were tested in three different scenarios: Precision, Speed, and Autonomy. Our proposed performance metric, *P*, Equation (5), was weighted and normalized. In *P*, 0 is the minimum, and 1 is the maximum. The DNN-based BC algorithm showed the best performance among others, with a performance *P* value of 0.92 in the *Precision* and *Autonomy* scenarios. The RTAB-Map algorithm showed the best *P* value with 0.96 when *Speed* is more important than others. In a case where *Autonomy* has a higher priority, Gmapping also showed a comparable performance value of 0.92 with the DNN-based BC. We believe that this comparative study on simulated outdoor navigations could be a useful tool for agricultural automation. 

## Figures and Tables

**Table 1:** Overview of the SLAM algorithms and the DNN-based BC along with their respective sensors and navigation mechanisms.

Algorithms	Sensors	Map	Navigation
GMapping [3]	LiDAR	Yes	AMCL
Cartographer [4]	LiDAR	Yes	AMCL
RTAB-Map [5]	Camera	Yes	RTAB-Map
DNN-based BC [6]	Camera	No	DNN

**Table 2:** Performance results in Driving Deviation (DD), Completion Time (CT), and Autonomy (AT) for GMapping, Cartographer, RTAB-Map, and DNN-based BC. The navigation from left-to-right (LR) and from right-to-left (RL) are calculated using min-max normalization. The Table also shows the actual standard deviation of driving deviation (D) in meters (m), the average completion time (C) in seconds (s), and the average human intervention in each SLAM algorithm and DNN-based BC for LR and RL. Note that bold number means minimum or maximum value for the regarding algorithm, and the up and down arrows mean better performance with bigger and smaller number, respectively.

Algorithms	Driving Deviation: DD (m) ↓		Completion Time: CT (s) ↓		Autonomy: AT ↑	
	LR	RL	LR	RL	LR	RL
GMapping	35.17 (±0.36)	35.03 (±0.51)	99.97 (±18.24)	99.88 (±34.20)	98.79% (±1)	**100.00% (±0)**
Cartographer	35.25 (±0.50)	35.35 (±0.72)	133.17 (±63.60)	96.16 (±29.70)	96.26% (±3)	95.00% (±4)
RTAB-Map	34.62 (±0.41)	34.29 (±0.40)	**99.47 (±37.32)**	**94.16 (±32.79)**	96.38% (±3)	92.35% (±6)
DNN-based BC	**34.45 (±0.25)**	**34.22 (±0.41)**	237.99 (±16.19)	239.54 (±19.36)	**100.00% (±0)**	**100.00% (±0)**

**Table 3:** Normalized performance results for Driving Deviation ($\tilde{DD}$), Completion Time ($\tilde{CT}$), and Autonomy for GMapping, Cartographer, RTAB-Map, and DNN-based BC. The navigation from left to right (LR) and from right to left (RL) are calculated using min-max normalization. Note that bold number means minimum or maximum value for the regarding algorithm, and the up and down arrows mean better performance with bigger and smaller number, respectively.

Algorithms	Normalized DD: $\tilde{\mathrm{DD}}$ ↓		Normalized CT: $\tilde{\mathrm{CT}}$ ↓		Autonomy: AT ↑	
	LR	RL	LR	RL	LR	RL
GMapping	0.90	0.71	0.004	0.04	98.79%	**100.00%**
Cartographer	**1.00**	1.00	0.24	0.01	96.26%	95.00%
RTAB-Map	0.21	0.06	**0.00**	**0.00**	96.38%	92.35%
DNN-based BC	**0.00**	**0.00**	1.00	1.00	**100.00%**	**100.00%**

**Table 4:** Overview of the scenarios where weighted performances based on different cases like precision driving, autonomy, and faster completion time. Note: the value of $\omega_{DD}$, $\omega_{CT}$, and $\omega_{AT}$ can be changed on your purpose as long as their sum is 1. Note that bold number means minimum or maximum value for the regarding algorithm, and the up arrow means better performance with bigger number.

Scenario	Weights ($\omega_{\mathrm{DD}},\omega_{\mathrm{CT}},\omega_{\mathrm{AT}}$)	Algorithms	Weighted Performance: *P* ↑
Precision	0.85, 0.075, 0.075	Gmapping	0.23
		Cartographer	0.13
		RTAB-Map	0.87
		DNN-based BC	**0.92**
Speed	0.075, 0.85, 0.075	Gmapping	0.93
		Cartographer	0.72
		RTAB-Map	**0.96**
		DNN-based BC	0.15
Autonomy	0.075, 0.075, 0.85	Gmapping	**0.92**
		Cartographer	0.87
		RTAB-Map	0.83
		DNN-based BC	**0.92**

## Data Availability

The source code presented in this study is openly available in [36].

## Abbreviations

The following abbreviations are used in this manuscript:

SLAM	Simultaneous Localization and Mapping
DNN	Deep Neural Network
CNN	Convolutional Neural Network
BC	Behavior Cloning
AMCL	Adaptive Monte Carlo Localization
ROS	Robot Operating System
